# Clinical Utility of Germline Whole-Exome Sequencing Beyond Multigene Panels in Hereditary Cancer

**DOI:** 10.3390/genes17091102

**Published:** 2026-09-11

**Authors:** Anastasia Dell’Elice, Lucia Lombardi, Federico Anaclerio, Claudia Palmarini, Nicole Canale, Lorenzo Secondi, Gregorio Stratta, Marco Vitali, Maria Saveria Tavoletta, Simona Grossi, Alessandra Babore, Cristina Milillo, Melania Dovizio, Patrizia Ballerini, Valentina Gatta, Liborio Stuppia, Ivana Antonucci

**Affiliations:** 1 Center for Advanced Studies and Technology (CAST), “G. d’Annunzio” University of Chieti-Pescara, 66100 Chieti, Italy; anastasia.dellelice@studenti.unich.it (A.D.); lucia.lombardi@unich.it (L.L.); claudia.palmarini@studenti.unich.it (C.P.); cristina.milillo@unich.it (C.M.); melania.dovizio@unich.it (M.D.); patrizia.ballerini@unich.it (P.B.); v.gatta@unich.it (V.G.); stuppia@unich.it (L.S.); 2 Department of Neurosciences, Imaging and Clinical Sciences, “G. d’Annunzio” University of Chieti-Pescara, 66100 Chieti, Italy; 3 Department of Human Sciences, Law, and Economics, Telematic University “Leonardo Da Vinci” (UNIDAV), Torrevecchia Teatina, 66100 Chieti, Italy; 4 Breast Unit, P.O. “G. Bernabeo”, 66026 Ortona, Italy; canalenicole@gmail.com (N.C.); secondilorenzo.md@gmail.com (L.S.); senologia@unich.it (G.S.); marco.vitali@gmail.com (M.V.); tavoletta.saveria@gmail.com (M.S.T.); sgrossi@unich.it (S.G.); 5 Department of Psychological, Health and Territory Sciences, “G. d’Annunzio” University of Chieti-Pescara, 66100 Chieti, Italy; alessandra.babore@unich.it; 6 Department of Innovative Technologies in Medicine and Dentistry, “G. d’Annunzio” University of Chieti-Pescara, 66100 Chieti, Italy

**Keywords:** whole-exome sequencing, hereditary cancer, precision oncology, genetic counselling

## Abstract

Germline Whole-Exome Sequencing (WES) has emerged as a powerful genomic approach for investigating Hereditary Cancer predisposition through the comprehensive analysis of coding regions across the genome. Although multigene panels currently represent the standard diagnostic approach for Hereditary Cancer assessment, a substantial proportion of high-risk individuals and families remain molecularly unexplained. In this setting, germline WES may serve as a valuable second-tier strategy by identifying pathogenic variants in genes not routinely included in conventional testing panels and by addressing part of the unresolved “missing heritability” observed across Hereditary Cancer syndromes. Increasing evidence supports its application in hereditary breast and ovarian cancer, Lynch-like syndrome, colorectal polyposis, hereditary diffuse gastric cancer, and ovarian cancer predisposition, where WES has contributed to the identification of additional susceptibility genes and improved molecular characterization. Beyond Hereditary Cancer diagnostics, exome-based approaches have also been explored for tumour profiling, homologous recombination deficiency (HRD) assessment, biomarker discovery, and therapeutic stratification. However, despite its significant potential, the clinical implementation of WES remains challenging because of the high burden of variants of uncertain significance (VUS), difficulties in variant interpretation, incidental findings, and the need for robust functional validation of candidate genes. This review critically examines the current evidence supporting the clinical utility of germline WES in Hereditary Cancer syndromes, focusing on the clinical scenarios in which WES may provide meaningful additional information beyond multigene panels, its diagnostic yield, limitations, and future perspectives in precision oncology.

## 1. Introduction

Genetic testing has profoundly transformed the management of hereditary cancers (HCs), improving risk assessment, surveillance strategies, clinical management, and preventive care for affected individuals and at-risk relatives [1,2]. The identification of pathogenic germline variants enables personalized therapeutic and surgical interventions while supporting predictive testing and risk-reduction strategies within families [3,4,5]. Accurate identification of individuals with inherited cancer susceptibility therefore represents a fundamental component of modern precision oncology and relies on both the characterization of cancer-associated genes and the appropriate selection of patients for genetic testing [6]. HCs are characterized by marked genetic heterogeneity, frequently involving multiple genes with variable penetrance and phenotypic expression [7,8]. Consequently, many patients with strong personal or familial cancer histories still receive negative or inconclusive genetic results, potentially leading to suboptimal surveillance strategies and inadequate clinical management. The advent of Next-Generation Sequencing (NGS) technologies has revolutionized cancer genetics by enabling the simultaneous analysis of multiple susceptibility genes through multigene panels (MGPs), which are now widely integrated into routine clinical practice [3]. These panels have significantly improved the detection of pathogenic variants (PVs) associated with HC predisposition and facilitated the implementation of personalized prevention and management strategies. International guidelines, including those from the National Comprehensive Cancer Network (NCCN) and the American Society of Clinical Oncology (ASCO), have progressively incorporated multigene testing into HC management algorithms [9]. In parallel, the American College of Medical Genetics and Genomics (ACMG) has developed a curated list of clinically actionable genes for the reporting of secondary findings identified during clinical exome and genome sequencing performed for other diagnostic indications [10]. These recommendations are intended to guide the reporting of secondary findings and should not be interpreted as a gene list for primary diagnostic testing or population screening. Nevertheless, despite their clinical utility, MGPs remain restricted to predefined gene sets and require continuous updating and validation as novel susceptibility genes emerge.

Despite the widespread adoption of multigene panels as the current standard approach for Hereditary Cancer assessment, a substantial proportion of high-risk individuals and families remain molecularly unexplained. Indeed, in a large cohort of approximately 165,000 individuals referred for hereditary cancer multigene panel testing, pathogenic/likely pathogenic variant detection rates ranged from 5.5% to 10.8%, depending on panel size [11]. Similarly, in a clinically selected cohort of unaffected individuals with a strong family history of cancer, pathogenic/likely pathogenic variants were identified in only approximately 5% of subjects [5], highlighting the substantial proportion of individuals and families that remain molecularly unexplained after conventional panel testing. This unresolved fraction, often referred to as “missing heritability”, represents one of the major challenges in cancer genetics. This concept is further supported by large population-based exome studies. Using germline Whole-Exome Sequencing data from the UK Biobank, Guan et al. developed a novel context-based variant aggregation approach and demonstrated that rare coding variants significantly contribute to cancer risk prediction beyond established susceptibility genes. These findings suggest that exome-wide analyses may help capture part of the inherited cancer risk that remains unexplained by conventional panel-based testing and provide further evidence for the role of WES in addressing missing heritability [12].

**Figure 1:**
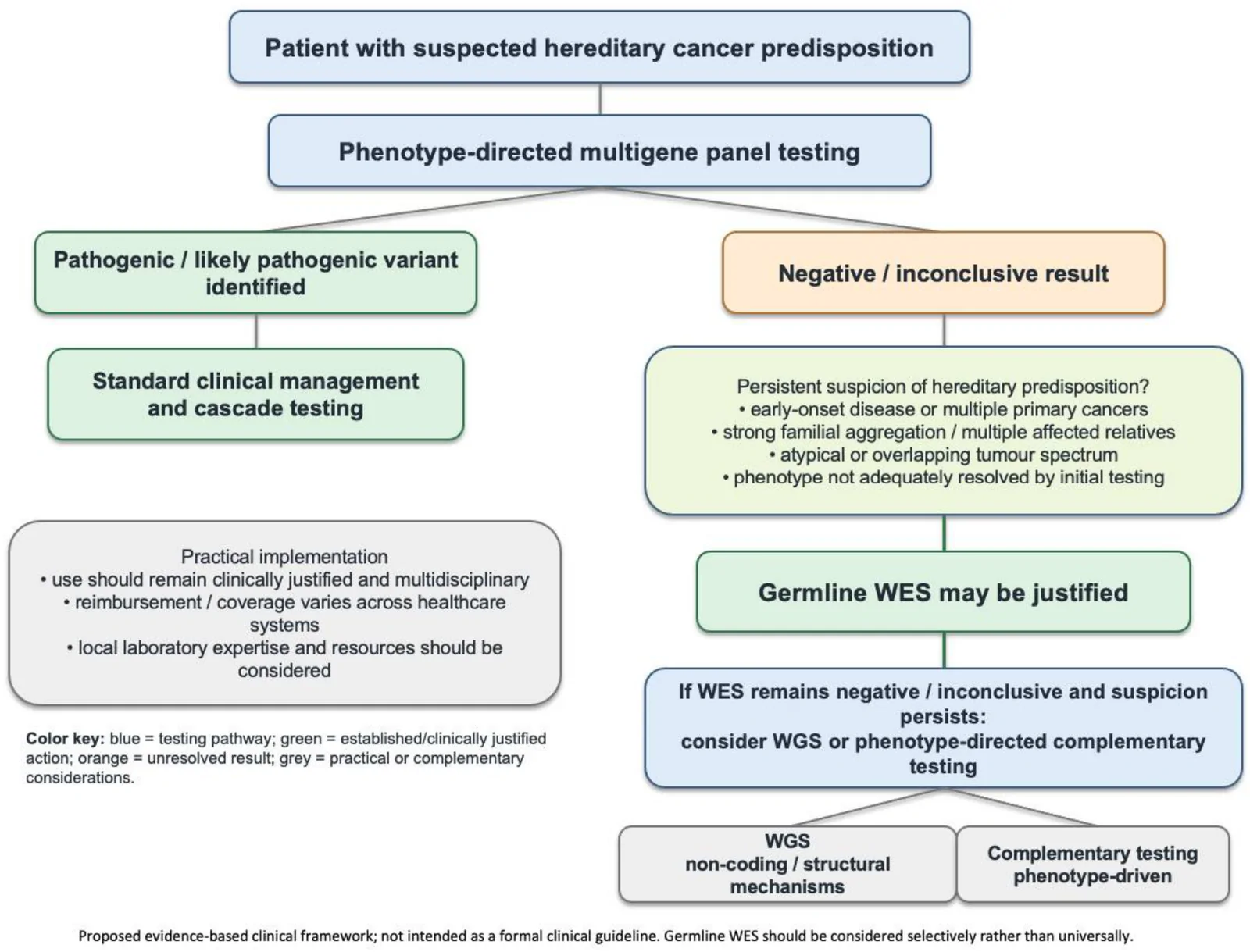
Clinical indications for germline WES beyond multigene panels in Hereditary Cancer.

WES is a next-generation sequencing approach that analyses the protein-coding regions of the inherited genome to identify pathogenic variants associated with inherited disease susceptibility. In contrast, somatic sequencing analyses DNA derived from tumour tissue to detect acquired mutations involved in tumour development, progression, and therapeutic response. In this setting, germline WES has emerged as a potential second-tier strategy capable of identifying pathogenic variants in genes not routinely included in diagnostic panels and of addressing part of the missing heritability observed across Hereditary Cancer syndromes. By enabling the comprehensive analysis of all protein-coding regions of the genome, WES provides broader genomic coverage compared with targeted panels and facilitates the identification of both established and novel cancer predisposition genes [13]. Compared with panel-based sequencing, WES also offers the advantage of a standardized platform that does not require continuous redesign as new susceptibility genes are discovered [14,15,16]. However, despite its significant potential, the clinical utility of WES beyond currently available multigene panels remains incompletely defined. This review therefore focuses on the clinical utility of germline WES in Hereditary Cancer syndromes, with particular attention to the clinical scenarios in which WES may provide meaningful additional information beyond conventional multigene panels. We discuss the current evidence regarding its diagnostic yield, contribution to unresolved Hereditary Cancer risk, limitations, and emerging applications in precision oncology (Figure 1).

### Methodology

This narrative review was conducted through a structured literature search of PubMed, Scopus, and Web of Science databases. The search included articles published from January 2015 to June 2026 using combinations of the following keywords: whole-exome sequencing, germline Whole-Exome Sequencing, Hereditary Cancer, cancer predisposition, multigene panel, clinical exome sequencing, Lynch syndrome, hereditary breast and ovarian cancer, genetic counselling, variants of uncertain significance, and incidental findings. Additional relevant publications were identified through manual screening of the reference lists of selected articles. Original research articles, systematic reviews, meta-analyses, and clinical guidelines published in English were considered. Studies were selected based on their relevance to the clinical applications, diagnostic yield, genetic counselling, ethical implications, and future perspectives of germline whole-exome sequencing in hereditary cancer. As this is a narrative review, no formal systematic review protocol or quality assessment was applied.

## 2. Applications of WES in Hereditary Cancer Syndromes

WES enables the comprehensive analysis of all protein-coding regions of the genome and facilitates the identification of clinically relevant germline variants associated with Hereditary Cancer predisposition. In individuals with a strong clinical suspicion of Hereditary Cancer who remain molecularly unexplained after multigene panels, germline WES may represent a valuable second-tier strategy for identifying additional susceptibility variants and addressing part of the missing heritability observed across Hereditary Cancer syndromes (Table 1). Given the broad and continuously expanding spectrum of hereditary cancer predisposition genes, Table 1 is not intended to provide an exhaustive catalogue of hereditary cancer syndromes. Rather, it summarizes selected clinical contexts in which germline WES has been specifically investigated and may provide additional information beyond conventional multigene panel testing. The application of WES has significantly expanded the understanding of hereditary breast cancer (HBC), contributing to the identification of more than 20 susceptibility genes, including *TP53*, *PALB2*, *PTEN*, *ATM*, and *CHEK2* [17]. Nevertheless, many familial breast cancer cases remain genetically unexplained, suggesting the involvement of additional low-penetrance or rare susceptibility genes. Early WES studies performed in BRCA-negative hereditary breast cancer families identified variants in genes associated with rare cancer syndromes, although no definitive causative variants were established because of limited cohort size and the inability of WES to detect intronic or regulatory alterations [18]. Subsequent studies further demonstrated the utility of WES in hereditary breast and ovarian cancer. Felicio et al. analyzed 52 Brazilian women at high risk for hereditary breast and ovarian cancer who tested negative for *BRCA1*, *BRCA2*, and *TP53* mutations, identifying 19 pathogenic or likely pathogenic variants across 18 genes, including *CHEK2*, *RAD51C*, *PMS2*, and *MUTYH*, together with alterations in non-canonical genes such as *FAN1*, *POLQ*, *RAD54L*, *DROSHA*, and *SLC34A2* [19]. Similarly, Van Tung et al. identified 56 variants across 37 cancer-related genes in 105 Vietnamese breast cancer patients, including alterations in *BRCA1*, *BRCA2*, *ATM*, *MLH1*, *PMS2*, *MSH2*, *MSH6*, *RAD54L*, and *RECQL* [20]. More recently, Qutob et al. investigated three Palestinian BRCA1/2-negative families with suspected hereditary breast cancer using germline WES and identified segregating variants in *RAD50*, *PRKAR1A*, *MSH4*, and *STAT6*. Although these findings require further functional validation and larger replication studies, they further illustrate the potential of germline WES to identify novel candidate susceptibility genes in unresolved hereditary cancer families [21]. Collectively, these studies suggest that germline WES may increase the diagnostic yield in selected BRCA-negative families by identifying pathogenic variants in genes not routinely included in conventional testing panels. Beyond breast cancer, germline WES has shown important applications across several Hereditary Cancer syndromes. In hereditary diffuse gastric cancer (HDGC), WES studies identified several candidate susceptibility genes, including *MUC4*, *ABCA13*, *ZNF469*, *FCGBP*, *IGFN1*, *RNF213*, and *SSPO*. However, the contribution of these genes to hereditary cancer predisposition remains preliminary and requires further functional validation and independent replication before they can be considered established susceptibility genes [22]. In colorectal polyposis, Dos Santos et al. identified pathogenic or likely pathogenic variants in 44.4% of patients negative for *APC* and *MUTYH* mutations, involving multiple genes associated with DNA repair and Hereditary Cancer predisposition [23]. Similarly, in Lynch-like syndrome (LLS), WES identified potentially pathogenic variants in approximately 35% of patients fulfilling Lynch syndrome criteria but lacking mismatch repair gene alterations [24]. These observations indicate that WES may be particularly useful in patients with unresolved hereditary colorectal cancer phenotypes after negative testing of the major susceptibility genes, where additional clinically relevant variants may be identified. Important contributions of germline WES have also emerged in ovarian cancer research. Zhu et al. identified several candidate susceptibility genes, including *TTC28*, *VPS13B*, *COL6A3*, *FREM2*, and *ANKRD11*. These findings represent exploratory associations that require additional functional and clinical validation before their role in hereditary ovarian cancer predisposition can be considered established [25]. More recently, a large WES study involving 2573 ovarian cancer patients and 13,923 controls confirmed the role of established susceptibility genes such as *BRCA1*, *BRCA2*, *BRIP1*, *RAD51C*, *RAD51D*, *MSH6*, and *PALB2*, while also identifying HELB as a novel candidate susceptibility gene associated with non-high-grade serous ovarian cancer [26]. Additional candidate genes, including *OR2T35*, *MYO1A*, *GABRP*, and *MIGA1*, further emphasized the genomic complexity underlying ovarian cancer predisposition. Germline WES has also been investigated in the context of hereditary hematologic malignancies (HHMs), an increasingly recognized and genetically heterogeneous group of cancer predisposition disorders. In patients with a strong personal or familial onco-hematological history who remain genetically unresolved, WES may provide broader assessment beyond predefined gene panels and facilitate the identification of both established and candidate susceptibility genes. Andrés-Zayas et al. [27] applied WES to 16 individuals with a strong personal or familial history of hematologic malignancies, identifying clinically relevant germline variants in cancer-related genes as well as novel candidate susceptibility genes, including *NFATC2* and *TC2N* [10]. These findings support the potential role of WES in the molecular characterization of selected families with unexplained hereditary hematologic malignancies. The identification of germline predisposition in hematologic malignancies also has specific clinical implications, including genetic counselling, familial testing, and, in selected patients undergoing allogeneic transplantation, donor selection [28]. Importantly, candidate-gene discovery through WES should be clearly distinguished from clinically actionable findings. Variants in genes lacking an established gene–disease association should not directly inform clinical management and require appropriate validation. Accordingly, the identification of novel candidate susceptibility genes should primarily be regarded as a research application of WES rather than as an immediate clinical diagnostic outcome. The principal germline WES studies across Hereditary Cancer syndromes and their diagnostic yields are summarized in Table 2.

### WES Beyond Germline Testing: Therapeutic Stratification and HRD Assessment

Although the primary focus of this review is germline WES in Hereditary Cancer predisposition, Whole-Exome Sequencing has also been explored in tumour profiling and precision oncology applications. These approaches are distinct from germline testing but may provide complementary information for therapeutic stratification and biomarker assessment. For example, He et al. performed paired tumour–normal WES in 47 patients with pancreatic ductal adenocarcinoma (PDAC), identifying recurrent somatic alterations in KRAS, TP53, SMAD4, ARID1A, and CDKN2A, together with deleterious germline variants in ATM, WRN, and PALB2 [29]. The study also reported novel gene fusions and correlations between genomic alterations, tumour mutational burden, and PD-L1 expression, highlighting the potential role of WES in precision oncology and therapeutic stratification. Similarly, Chiang et al. demonstrated the utility of tumour-based WES for homologous recombination deficiency (HRD) assessment in epithelial ovarian cancer. WES-derived HRD scores showed strong concordance with standard HRD assays and were associated with platinum sensitivity and improved progression-free survival, supporting the use of exome-based approaches for treatment stratification and prediction of response to PARP inhibitors [30].

## 3. Genetic Counselling Challenges in Germline WES

The implementation of germline WES in Hereditary Cancer practice has substantially increased the complexity of genetic counselling because it enables the simultaneous identification of a large number of genetic variants with heterogeneous clinical significance. Compared with conventional multigene panels, WES generates a broader spectrum of findings, including variants in genes with emerging evidence of cancer predisposition, variants of uncertain significance (VUSs), and incidental findings unrelated to the primary indication for testing [31]. As in other clinical applications of exome sequencing, comprehensive pre-test and post-test genetic counselling represents an essential component of germline WES, ensuring informed consent, appropriate interpretation of results, and discussion of uncertain or unexpected findings. In the context of Hereditary Cancer, counselling should additionally address cancer-specific implications for surveillance, risk-reducing strategies, and at-risk relatives. A major challenge in Hereditary Cancer genetics is the interpretation and communication of VUSs and candidate susceptibility genes for which clinical validity remains incompletely established. Patients should be informed that variant classifications may evolve over time as additional evidence becomes available and that periodic re-evaluation of genomic findings may therefore be necessary. Variants previously considered pathogenic may be reclassified, whereas VUSs may eventually acquire clinical significance, highlighting the importance of long-term follow-up and reanalysis of genomic data [32]. The identification of incidental or secondary findings represents an additional challenge because these findings may have important psychological and ethical implications for patients and their relatives, including increased anxiety and decisional uncertainty [33]. These aspects are particularly relevant in hereditary cancer, where genetic results may directly influence recommendations regarding intensified surveillance, prophylactic surgery, and targeted therapeutic strategies for both affected individuals and at-risk relatives [4,5,11,12]. Consequently, pre-test counselling should include a thorough discussion regarding the possibility of secondary findings and incorporate patient preferences concerning their disclosure. Unlike many other clinical applications of genomic testing, germline WES in Hereditary Cancer has implications that extend beyond the individual patient. The identification of a pathogenic variant may facilitate predictive testing in at-risk relatives, support cascade testing, and enable personalized surveillance and risk-reduction strategies within families. In selected situations, genetic findings may also influence reproductive decision-making, including discussions regarding reproductive options and pre-implantation genetic testing. Overall, although germline WES offers important opportunities to improve the molecular diagnosis of Hereditary Cancer syndromes, its increasing complexity highlights the need for multidisciplinary management involving geneticists, oncologists, genetic counsellors, psychologists, and other healthcare professionals. Standardized variant interpretation, clear communication of results, and appropriate long-term follow-up remain essential to ensure that genomic information is translated into meaningful, responsible, and patient-centred clinical care [34,35].

## 4. Psychological and Ethical Implications of WES

WES may have multifaceted psychological repercussions for individuals and their families, affecting emotional well-being, self-perception, and decision-making processes. The disclosure of genetic results may increase anxiety, fear, or psychological distress, especially when findings indicate a predisposition to severe, chronic, or currently untreatable diseases [36]. Uncertainty plays a central role in this context, as variants of unknown significance (VUS can generate confusion, frustration, and persistent worry due to the lack of clear clinical interpretation or actionable outcomes [37,38]. Individuals may also experience anticipatory anxiety related to potential future illness, leading to hypervigilance toward bodily symptoms and changes in life planning [39]. WES results can affect personal and familial identity, as genetic risk information may reshape how individuals perceive themselves and their relatives, sometimes inducing feelings of guilt, blame, or moral responsibility toward family members who may share the same genetic variants [40]. Among the most psychologically challenging aspects of WES are incidental or secondary findings, which arise independently of the original clinical question that motivated testing and often involve conditions with serious health implications [41]. Since individuals typically undergo WES seeking answers for a specific medical concern, the unexpected disclosure of additional genetic risks can increase anxiety and psychological stress [42]. These findings may indicate a predisposition to adult-onset disorders, including HCs or cardiovascular diseases; awareness of such risks can undermine an individual’s sense of control and perceived health status, transforming someone who previously considered themselves healthy into a “patient-in-waiting” [39]. In addition, individuals may feel a responsibility to inform at-risk relatives, a process that can generate guilt, fear of stigma, or strain within family relationships [40]. For these reasons, professional guidelines strongly emphasize the role of informed consent and genetic counselling in preparing individuals for the possibility of incidental findings, helping them contextualize risks, manage emotional reactions, and make meaning of unexpected genetic information [41,42]. Nevertheless, a study based on patients’ interviews highlighted that the return of WES results to healthy individuals is usually well tolerated and often perceived as valuable, although concerns may arise in specific circumstances, such as the identification of susceptibility to Alzheimer’s disease. The authors highlight that the broader impact and clinical utility of returning results on a large scale remain uncertain and warrant further research [43]. In the reproductive context, WES outcomes may influence decisions regarding family planning, potentially generating decisional conflict or emotional burden [44]. Consequently, the literature emphasizes the importance of pre- and post-test genetic counselling to support emotional adjustment, promote informed understanding, and mitigate adverse psychological effects associated with WES [43]. Additionally, psychological support at the time of result disclosure and during subsequent decision-making processes could be beneficial in helping individuals cope with the emotional impact of findings and integrate genetic information into health-related choices. In hereditary cancer, these psychological and ethical issues are further amplified by the familial nature of inherited cancer predisposition. Decisions regarding predictive testing of relatives, cascade testing, intensified surveillance, prophylactic interventions, and reproductive planning may have profound implications not only for the individual undergoing testing but also for multiple family members. Consequently, multidisciplinary management involving clinical geneticists, oncologists, psychologists, and genetic counsellors is essential to support informed decision-making and facilitate appropriate long-term follow-up.

## 5. Advantages and Limitations of Germline WES in Hereditary Cancer

Although germline WES offers broader genomic coverage than conventional multigene panels and may improve the diagnostic yield in unresolved Hereditary Cancer cases, its clinical implementation is also associated with important technical and interpretative limitations [45]. Because it interrogates primarily coding regions, clinically relevant variants located in deep intronic, regulatory, or other non-coding regions may remain undetected. In addition, WES has reduced sensitivity for the detection of certain classes of genetic alterations, including copy number variations, repeat expansions, structural rearrangements, and low-level mosaicism. Complementary molecular techniques may therefore be required in selected clinical scenarios [46]. Another important challenge concerns genomic interpretation. Although WES may identify additional candidate susceptibility genes, many detected variants remain difficult to classify because of limited functional evidence, incomplete genotype–phenotype correlations, and evolving knowledge regarding gene–disease associations. Consequently, multidisciplinary expertise, standardized interpretation frameworks, and periodic reanalysis of genomic data remain essential for the accurate clinical application of WES [46]. Economic and reimbursement considerations remain important determinants of the clinical implementation of germline WES. Coverage and reimbursement vary across healthcare systems and clinical indications, and the additional costs associated with bioinformatic analysis, variant interpretation, data storage, and multidisciplinary expertise should be considered alongside sequencing costs. Recent health-economic evidence suggests that WES and WGS may be cost-effective as diagnostic tools in selected clinical settings; however, cost-effectiveness varies substantially according to the clinical context, patient population, and healthcare system, and evidence specifically addressing hereditary cancer predisposition remains limited [47]. Therefore, economic considerations currently support selective, clinically justified use of WES rather than its universal implementation. Overall, germline WES represents a valuable complementary genomic approach for Hereditary Cancer evaluation. However, its optimal integration into clinical practice requires appropriate patient selection, rigorous variant interpretation, and continuous refinement of analytical and bioinformatic approaches to ensure clinically meaningful and patient-centred genomic care (Table 3).

## 6. Toward Universal Whole-Exome Sequencing in Hereditary Cancer?

Recent proposals have advocated the use of WES in all patients diagnosed with specific types of cancer or presenting with early-onset disease, such as colorectal cancer diagnosed before the age of 50 years, OC, or BC diagnosed before 35 years of age, regardless of personal or family history [48]. This “universal” germline testing approach aims to improve the detection of cancer predisposition variants that might otherwise remain unrecognized, particularly in individuals without evident high-risk clinical features. Despite its potential, however, the indiscriminate application of WES remains controversial. Although technically feasible, universal testing would impose substantial organizational and clinical burdens, including increased demand for genetic counselling and the growing complexity of genomic data interpretation [33]. In addition, current evidence supports the clinical utility of WES primarily for genes with established therapeutic or predictive relevance, where results can directly influence patient management and surveillance strategies [12]. Importantly, the application of WES to unselected populations is associated with a higher frequency of VUSs, often without a proportional increase in diagnostic yield [16]. This may complicate clinical decision-making, particularly in cases involving genes not typically associated with the observed tumour spectrum or lacking robust genotype–phenotype correlations. Taken together, while WES represents a powerful tool in Hereditary Cancer genetics, its universal application remains premature. Taken together, germline WES should currently be reserved for carefully selected unresolved Hereditary Cancer cases following negative multigene panels, rather than being applied universally.

## 7. Clinical Recommendations for the Implementation of WES

The implementation of germline WES in clinical oncology requires transparency in analytical methods, data interpretation, reporting strategies, and long-term storage of genomic information [48]. Standardized laboratory protocols and validated analytical procedures are essential to ensure consistency, regulatory compliance, and alignment with informed patient consent. At present, universal germline WES for HC predisposition assessment is not routinely recommended. Instead, WES should be applied selectively and guided by specific clinical indications and diagnostic hypotheses. More specifically, escalation from phenotype-directed multigene panel testing to germline WES may be justified when a negative or inconclusive panel result is accompanied by persistent clinical suspicion of hereditary cancer predisposition. Relevant clinical features may include early-onset disease, multiple primary cancers, strong familial aggregation, atypical or overlapping tumour spectra, and phenotypes suggesting genetic heterogeneity that is not adequately addressed by the initial testing strategy [10,49]. In these settings, WES should be regarded as a second-tier diagnostic approach rather than as a universal replacement for established multigene panels. Importantly, genomic sequencing should not be considered in isolation from phenotype-directed complementary investigations. In patients with clinical features suggestive of specific inherited cancer-predisposition disorders, functional or biomarker-based testing may provide information that cannot be obtained from WES alone. For example, telomere-length measurement by flow-FISH may be particularly informative when a telomere biology disorder is clinically suspected, especially in patients with bone-marrow failure, pulmonary fibrosis, liver disease, characteristic mucocutaneous abnormalities, or a suggestive family history. In this setting, telomere-length assessment should be considered complementary to germline sequencing and may help guide subsequent genetic investigation and variant interpretation [50]. Careful evaluation of personal and family history remains fundamental for identifying individuals most likely to benefit from testing. In selected clinical scenarios, particularly when therapeutic decision-making is required, molecular analysis may still be appropriate even in patients who do not fully meet established testing criteria, provided that the analysis focuses on genes with established predictive or therapeutic relevance. A targeted and hypothesis-driven strategy is therefore preferred, with stepwise analysis prioritizing clinically actionable genes. The inclusion of moderate-risk genes or genes not clearly related to the observed phenotype should be carefully evaluated and discussed with patients, emphasizing both potential benefits and interpretative uncertainties. Pre-test and post-test genetic counselling remain essential components of WES implementation to ensure informed decision-making, appropriate interpretation of results, and adequate patient support. Furthermore, clinicians prescribing WES should receive adequate genomic training to fully understand the broader implications of sequencing results and appropriately refer patients for specialized genetic evaluation [51]. Overall, the effective integration of WES into clinical oncology depends on multidisciplinary collaboration, standardized interpretative frameworks, and a balanced approach capable of maximizing clinical utility while minimizing uncertainty and inappropriate genomic interpretation. A practical framework outlining the main clinical scenarios in which germline WES may be considered is presented in Figure 1.

## 8. Discussion

The clinical role of germline WES in Hereditary Cancer is evolving rapidly as genomic technologies become increasingly integrated into routine oncology practice. Although multigene panels currently represent the standard approach for Hereditary Cancer assessment, a substantial proportion of individuals with a strong personal or family history of cancer remain molecularly unexplained. In this context, the available evidence suggests that germline WES may provide additional diagnostic value by identifying pathogenic variants in genes not routinely included in conventional testing panels and by contributing to the understanding of the missing heritability observed across Hereditary Cancer syndromes. The studies reviewed herein demonstrate that the diagnostic yield of germline WES varies considerably according to tumour type, patient selection criteria, and prior genetic testing. The greatest clinical utility appears to be observed in carefully selected individuals with a strong suspicion of Hereditary Cancer who remain negative after standard multigene panels. In these settings, WES may identify additional susceptibility genes, expand the spectrum of Hereditary Cancer predisposition, and improve molecular characterization. However, many candidate genes identified through exome-wide analyses still lack sufficient functional validation and robust genotype–phenotype correlations to support routine clinical implementation. The increasing use of germline WES also introduces important interpretative, ethical, and organizational challenges. High rates of variants of uncertain significance, the possibility of incidental findings, and the need for periodic reanalysis of genomic data require multidisciplinary expertise and comprehensive genetic counselling. Consequently, the clinical benefit of WES depends not only on sequencing technology but also on the availability of standardized interpretation frameworks and specialized Hereditary Cancer services. Current evidence does not support the routine use of universal germline WES in unselected cancer populations. Rather, a targeted and clinically guided approach remains the most appropriate strategy, with WES serving as a second-tier diagnostic tool in selected unresolved cases. Future advances in variant interpretation, functional genomics, international data-sharing initiatives, and large population-based exome studies may further clarify the role of WES in Hereditary Cancer risk assessment and precision oncology. Although germline WES currently represents a practical compromise between diagnostic yield, cost, and data interpretation, whole-genome sequencing (WGS) is emerging as a complementary technology with the potential to overcome several limitations of exome-based approaches. Compared with WES, WGS enables the detection of deep intronic variants, structural variants, copy number alterations, and regulatory changes that are not routinely captured by exome sequencing, although its broader implementation is currently limited by higher costs, increased data complexity, and greater analytical demands. From a practical perspective, WGS may therefore be considered in selected patients who remain molecularly unexplained after multigene panel testing and WES, particularly when a strong hereditary phenotype persists and a non-coding or structural mechanism is suspected [52,53]. However, escalation to WGS should also consider analytical and interpretative complexity, data-storage requirements, local laboratory expertise, healthcare resources, and reimbursement. Accordingly, WGS should currently be viewed as a complementary or subsequent genomic strategy in selected unresolved cases rather than as a routine replacement for WES. Furthermore, the integration of artificial intelligence-assisted variant interpretation, polygenic risk scores, multi-omics approaches, and functional genomics is expected to improve the interpretation of genomic findings and facilitate the identification of clinically relevant susceptibility variants in unresolved hereditary cancer cases. Overall, germline WES represents a valuable complement to conventional multigene panels rather than a replacement for it. Its greatest contribution currently lies in carefully selected patients with unresolved Hereditary Cancer phenotypes, where it may provide clinically meaningful information beyond existing diagnostic approaches.

## Figures and Tables

**Table 1:** Selected Hereditary Cancer Syndromes and Clinical Applications of WES.

Syndrome	Established Susceptibility Genes	Main Clinical Contribution of WES
HBOC	*BRCA1*, *BRCA2*, *PALB2*, *ATM*, *CHEK2*	Identification of additional susceptibility genes and unexplained hereditary risk
LLS	*MLH1*, *MSH2*, *MSH6*, *PMS2*, *EPCAM*	Investigation of missing heritability and non-canonical predisposition genes
CP	*APC*, *MUTYH*	Expansion of diagnostic yield through alternative DNA repair genes
HDGC	*CDH1*	Discovery of novel candidate susceptibility genes
OC	*BRCA1*, *BRCA2*, *BRIP1*, *RAD51C*, *RAD51D*, *PALB2*	Risk assessment, HRD characterization and therapeutic stratification
HHMs	*DDX41*, *RUNX1*, *GATA2*, *CEBPA*, *ETV6*, *ANKRD26*	Identification of germline predisposition variants in genetically heterogeneous or unresolved familial hematologic malignancies and potential identification of candidate susceptibility genes

**Table 2:** Diagnostic Yield, Main Findings, and Level of Evidence of Germline Whole-Exome Sequencing Studies in Hereditary Cancer Syndromes. All studies included were conducted on germline tissue by WES (Abbreviations: HBOC, hereditary breast and ovarian cancer; HDGC, hereditary diffuse gastric cancer; CP, colorectal polyposis; LLS, Lynch-like syndrome; OC, ovarian cancer; P/LP, pathogenic/likely pathogenic; NR, not reported).

Syndrome	Study	N.	Previous Testing	Diagnostic Yield	Main Findings	Level of Evidence
HBOC	Liu et al., 2022 [18]	3 families	Negative 64-gene panel	0%	Candidate genes (*UBASH3A*, *MYH13*, *UTP11L*, *PAX7*)	Exploratory candidate genes; no functional validation
HBOC	Felicio et al., 2021 [19]	52	BRCA1/BRCA2/TP53 negative	30.8% (16/52)	19 P/LP variants in 18 established cancer predisposition genes	Established susceptibility genes
HBOC	Van Tung et al., 2025 [20]	105	NR	39.1% (41/105)	10 P/LP variants including *BRCA1*, *BRCA2*, *PMS2*, *SLX4*, *RECQL* and *FAN1*	Established susceptibility genes
HBOC	Qutob et al., 2026 [21]	3 families	BRCA1/2 negative	NR	Candidate genes (*RAD50*, *PRKAR1A*, *MSH4*, *STAT6*)	Exploratory candidate genes; segregation evidence only
HDGC	Liu et al., 2022 [22]	284	CDH1-negative cases	NR	Candidate genes (*MUC4*, *ABCA13*, *ZNF469*, *FCGBP*, *IGFN1*, *RNF213*, *SSPO*)	Candidate genes requiring functional validation
CP	Dos Santos et al., 2025 [23]	27	APC/MUTYH negative	44.4% (12/27)	17 P/LP variants in DNA repair and cancer predisposition genes	Established susceptibility genes
LLS	Dos Santos et al., 2022 [24]	20	dMMR tumours; MMR genes negative	35.0% (7/20)	Eight P/LP variants in cancer predisposition genes	Established susceptibility genes
OC	Zhu et al., 2020 [25]	158	BRCA1/2 negative (86.5%) or untested	NR	Candidate genes (*TTC28*, *VPS13B*, *COL6A3*, *FREM2*, *ANKRD11*)	Exploratory candidate genes requiring further validation
OC	Dicks et al., 2025 [26]	2537 cases/13,923 controls	Mixed cohorts	NR	Confirmed established susceptibility genes; *HELB* identified as a novel candidate gene	Established genes confirmed; HELB represents an emerging susceptibility gene

**Table 3:** Advantages and Limitations of Germline Whole-Exome Sequencing in Hereditary Cancer.

Advantages of WES	Clinical Implications	Limitations of WES	Clinical Implications
Comprehensive coding region analysis	Simultaneous evaluation of protein-coding genes associated with Hereditary Cancer predisposition	Limited genomic coverage	Non-coding and regulatory variants may remain undetected
Detection of known and novel variants	Identification of established susceptibility genes and novel candidate genes	Reduced sensitivity for certain alterations	Limited detection of CNVs, repeat expansions, and mosaicism
Broader genomic coverage than multigene panels	May help explain unresolved missing heritability	Interpretative complexity	Requires advanced bioinformatic analysis and multidisciplinary expertise
Supports personalized medicine and precision oncology	Facilitates individualized surveillance and therapeutic strategies	Variants of uncertain significance (VUSs)	Many variants lack clear pathogenic interpretation
Useful in high-risk Hereditary Cancer settings	Particularly valuable in early-onset cancers and strong family histories	Incidental or secondary findings	May generate ethical and psychological challenges
Facilitates biomarker discovery and therapeutic stratification	Supports HRD testing and targeted therapies	Coverage and reimbursement vary across healthcare systems and clinical indications	Economic and reimbursement constraints may limit widespread implementation

## Data Availability

Data are available upon request.

## Abbreviations

The following abbreviations are used in this manuscript:

HCs	Hereditary Cancers
HCS	Hereditary Cancer Syndrome
OC	Ovarian Cancer
BC	Breast Cancer
HBOC	Hereditary Breast and Ovarian Cancer
WES	Whole-Exome Sequencing
MGPs	Multigene Panels
ASCO	American Society of Clinical Oncology
ACMG	American College of Medical Genetics
HRD	Homologous Recombination Deficiency
VUS	Variant of Uncertain Significance
PV	Pathogenic Variant
LPV	Likely Pathogenic Variant
NGS	Next-Generation Sequencing
NCCN	National Comprehensive Cancer Network
HDGC	Hereditary Diffuse Gastric Cancer
CNV	Copy Number Variation
PDAC	Pancreatic Ductal Adenocarcinoma
